# Direct observation of peptide hydrogel self-assembly†

**DOI:** 10.1039/d1sc06562a

**Published:** 2022-08-16

**Authors:** Zoë C. Adams, Erika J. Olson, Tania L. Lopez-Silva, Zhengwen Lian, Audrey Y. Kim, Matthew Holcomb, Jörg Zimmermann, Ramkrishna Adhikary, Philip E. Dawson

**Affiliations:** Department of Chemistry, The Scripps Research Institute 10550 North Torrey Pines Road La Jolla California 92037 USA dawson@scripps.edu; Chemical Biology Laboratory, National Cancer Institute, National Institutes of Health Frederick MD 21702 USA

## Abstract

The characterization of self-assembling molecules presents significant experimental challenges, especially when associated with phase separation or precipitation. Transparent window infrared (IR) spectroscopy leverages site-specific probes that absorb in the “transparent window” region of the biomolecular IR spectrum. Carbon–deuterium (C–D) bonds are especially compelling transparent window probes since they are non-perturbative, can be readily introduced site selectively into peptides and proteins, and their stretch frequencies are sensitive to changes in the local molecular environment. Importantly, IR spectroscopy can be applied to a wide range of molecular samples regardless of solubility or physical state, making it an ideal technique for addressing the solubility challenges presented by self-assembling molecules. Here, we present the first continuous observation of transparent window probes following stopped-flow initiation. To demonstrate utility in a self-assembling system, we selected the MAX1 peptide hydrogel, a biocompatible material that has significant promise for use in drug delivery and medical applications. C–D labeled valine was synthetically introduced into five distinct positions of the twenty-residue MAX1 β-hairpin peptide. Consistent with current structural models, steady-state IR absorption frequencies and linewidths of C–D bonds at all labeled positions indicate that these side chains occupy a hydrophobic region of the hydrogel and that the motion of side chains located in the middle of the hairpin is more restricted than those located on the hairpin ends. Following a rapid change in ionic strength to initiate self-assembly, the peptide absorption spectra were monitored as function of time, allowing determination of site-specific time constants. We find that within the experimental resolution, MAX1 self-assembly occurs as a cooperative process. These studies suggest that stopped-flow transparent window FTIR can be extended to other time-resolved applications, such as protein folding and enzyme kinetics.

## Abstract 2

To facilitate the characterization of phase-transitioning molecules, site-specific non-perturbative infrared probes are leveraged for continuous observation of the self-assembly of fibrils in a peptide hydrogel following stopped-flow initiation.

## Graphical abstract

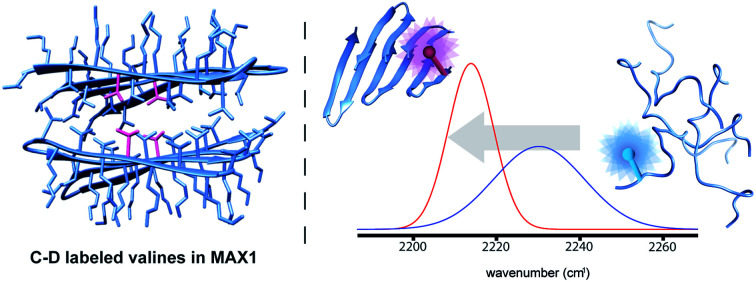

## Introduction

Self-assembling peptides can adopt a number of known structures including those in which the ordering of peptides into fibrils leads to the formation of three-dimensional hydrogels. These high water-content networks can be utilized to encapsulate and deliver therapeutics, proteins, and cells for drug delivery and tissue engineering. A better understanding of the formation of large peptide aggregates, often composed of distinct β-sheet structures, may also have implications for the study of the self-assembly processes leading to amyloid diseases and the development of useful biomimetic materials.^1^ As such, synthetic peptides with a tailored propensity to self-assemble can provide important insights into natural aggregation as well as the formation of designed materials.

**Scheme 1:**
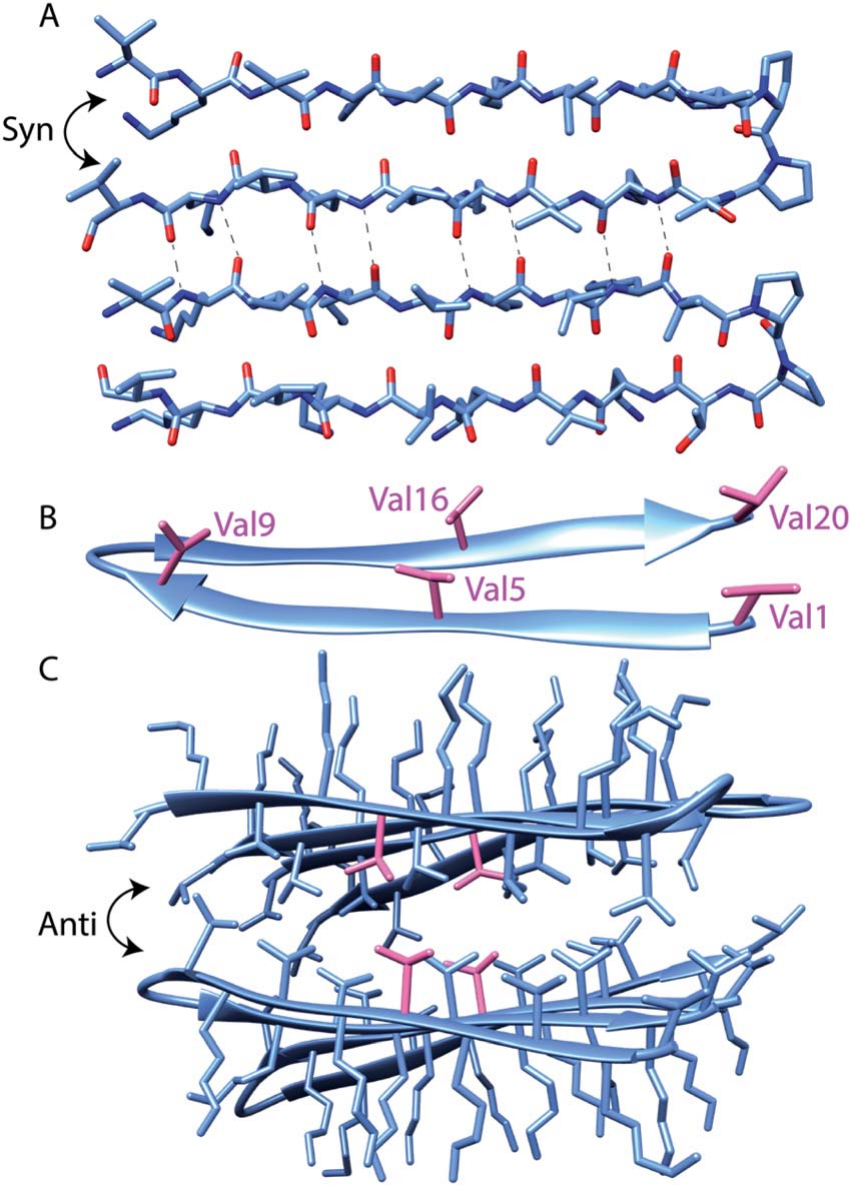
Assembly states of MAX1 Coordinates taken from solid-state NMR structure (PDB 2N1E) of MAX1 in fibrillar state. The sequence of MAX1, with positions at which labels are incorporated in bold, is **V**KVK**V**KVK**V**_D_PPTKVK**V**KVK**V**-NH_2_ (A): MAX1 peptide hairpin backbone conformation. Intermolecular hydrogen bonding between parallel (*syn*) β-hairpins is highlighted. (B): the locations of the five incorporated perdeuterated valine side chains (shown in magenta). (C): four MAX1 hairpins exemplify the assembled structure, with stacked anti hairpin planes creating a hydrophobic core and hydrophilic exterior. Val5 side chains are shown in magenta, as an example of C–D bond labeling to probe the core.

The MAX family of peptide hydrogels, developed by Schneider and co-workers, are clinically relevant biomaterials that emulate the characteristic amphiphilic β-sheet character of disease-implicated peptides like amyloid-β.^2^ Due to their tunable mechanical properties and biodegradability, MAX peptide hydrogels have been explored for therapeutic applications.^3^ Derivatives of MAX hydrogel peptides have been designed to undergo folding and self-assembly yielding hydrogels with varied materials properties depending on pH, salt concentration, buffer composition, and temperature.^3*e*,4^ The structure of the peptide assemblies has been analyzed by circular dichroism (CD), molecular dynamics simulations, and solid-state NMR, supporting a model in which the peptide adopts a β-hairpin structure with a type II' ^D^P^L^P β-turn.^5^ Interchain hydrogen bonding in the peptide backbone results in extended amphiphilic β-sheet filaments, which in turn pack together to desolvate the hydrophobic face and form a bilayered cross β-sheet structure (Scheme 1).^5*b*^ In this model, the valine side chains are fully buried in the hydrophobic core of each fibril while lysine side chains are solvent exposed on the exterior of the fibril. These fibrils subsequently form noncovalent nodes that are composed of extended intersecting structures, resulting in the macroscopic gel.^6^ Importantly, self-assembly of these peptides into hydrogels can be triggered by a simple increase in temperature or salt concentration.^4*d*,*e*,7^ Additionally, studies have shown a 1 : 1 mixture of MAX1 with its enantiomer, D-MAX1, results in co-assembled fibrils that form similar yet distinct hydrogels.^8^

**Scheme 2:**
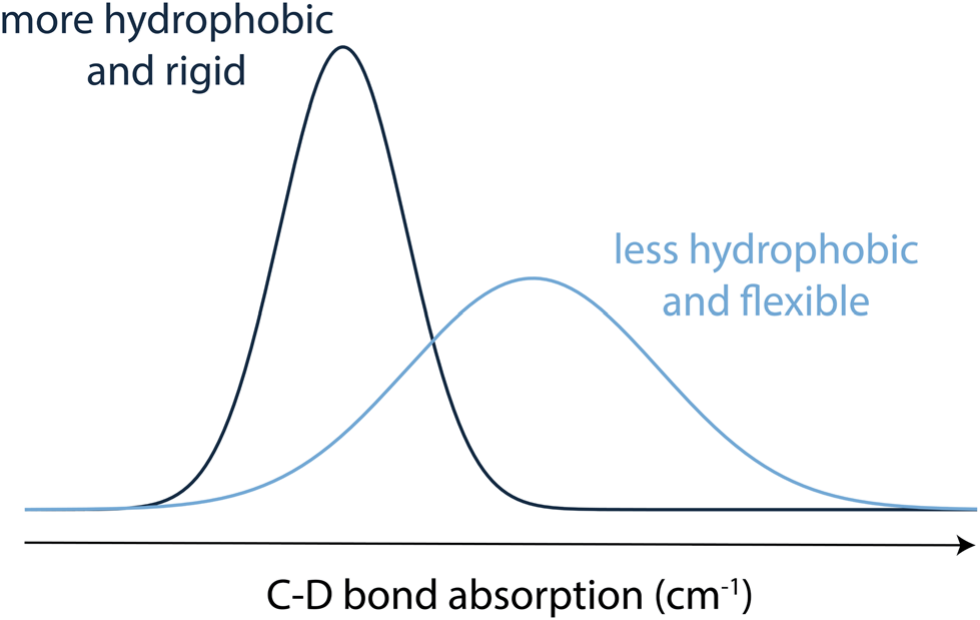
Schematic representation of IR absorption bands of C–D bonds as vibrational labels of a peptide. The peak frequency and line width depend on the polarity of the local environments and the flexibility of the peptide structure, respectively.

Phase transitions during the formation of self-assembling materials can be difficult to study since the assembly process can impede signal in techniques that rely on free motion of dissolved molecules, while techniques that measure macroscopic properties of the assembly often cannot characterize the fully solvated precursors.^9^ For example, while NMR is well suited for studying the precursors, gelation would result in a disappearance of signal over time, whereas ssNMR can only report on the final self-assembled state. Imaging through AFM or SEM can interrogate the final state but are insufficient for understanding the full transition. While rheology can be applied to the entire transition, it reports only on the mechanical properties of the hydrogel rather than more nuanced insights into molecular level interactions and peptide conformation. Similarly, while circular dichroism is a useful tool for peptide materials that adopt a secondary structure in addition to the macroscopic gel property, it may not be relevant for all assembling systems and cannot address interactions on the specific residue or atomic level. In contrast, vibrational spectroscopy has the advantage of directly measuring molecular vibrations, making it an ideal tool to characterize the structure of the peptide in any physical state.^10^ Although spectral crowding usually prevents the observation of individual bond vibrations in peptides and proteins, site-specific carbon–deuterium (C–D) bond labeling shifts the bond absorption to a transparent window (1800–2500 cm^−1^) in the IR spectra of proteins, facilitating characterization of local protein environments with high spatial as well as temporal resolution.^11^ C–D bonds have been employed to probe both soluble (monomeric) and aggregated (oligomeric) forms of proteins.^12^ Moreover, replacing C–H bonds in the backbone or side chains of a peptide with C–D bonds, can nonperturbatively probe secondary and tertiary structures of the protein, including residues in the hydrophobic core for which extrinsic IR probes cannot be used without significant perturbation.^11,13^ Prior studies have confirmed that C–D labelling does not significantly perturb the system compared to its native C–H moieties.^14^ Along with nonperturbative carbon–deuterium (C–D) bonds, other extrinsic transparent window IR probes including nitriles, azides, alkynes, and esters have facilitated characterization of local protein environments.^10*a*,15^ Importantly, C–D bond vibrations are sensitive to changes in peptide microenvironments.^10*a*,16^ The IR stretch absorption of C–D bonds shifts to red in a less solvated, more hydrophobic, environment and structural fluctuations result in broad IR absorption spectra, whereas narrower absorption spectra indicate a more rigid local environment (Scheme 2).^17^ Therefore, C–D bond IR probes are well suited to study self-assembling peptides such as MAX1.

While steady-state spectra of IR probe-labeled self-assembling peptides can provide important structural insights into the properties of the assembled and unassembled state, time-resolved experiments can capture the spectral evolution during the assembly and identify potential intermediate conformations of the peptides on the assembly pathway. Stopped-flow mixing with rapid-scan FTIR detection (SF-FTIR), can be used to trigger a kinetic process by initiating a rapid change in sample conditions followed by time-resolved detection. In previous work, SF-FTIR of intrinsic IR chromophores, such as the amide group vibrations of the backbone, has been used for the study of global protein folding, binding, and enzymatic reactions.^18^ In contrast, site-specific probes have yet to be applied to SF-FTIR of such complex biomolecular processes.

Herein, the steady-state conformation and kinetics of self-assembly of MAX1 is investigated by C–D bonds in conjunction with SF-FTIR. Using C–D bonds as site-specific IR probes enables detection of local variation in polarity and conformational heterogeneity within the hairpin. To estimate the site-specific assembly-induced changes in the IR absorption spectra of the probe, we synthesized a set of five MAX1 peptides, each perdeuterated at a specific valine residue in the sequence since the formation of self-assembled fibrils is understood to be primarily influenced by the formation of the valine rich hydrophobic core. We then collected steady-state spectra of the monomer and assembled states. Based on the magnitude of the local spectral shifts upon assembly, four sites were selected for kinetic studies using SF-FTIR. We find that residues in the middle of the MAX1 β-hairpin undergo the greatest change in local environment upon assembly and the time-resolved data fit a single exponential.

## Experimental methods

### Synthesis of MAX1 peptides

The nondeuterated and deuterated variants of MAX1 were synthesized on a CS Bio Automated Peptide Synthesizer (model CS336X) using Fmoc solid-phase peptide synthesis (SPPS) on 0.1 or 0.2 mmol TentaGel Rink Amide XV resin. Fmoc deprotection from the peptide-resin was achieved by treatment with 20% 4-methyl piperidine, 1% 1,8-diazabicyclo[5.4.0]undec-7-ene, and 79% DMF (v/v/v) for a total of 10 min. Five equivalents of nondeuterated amino acids were coupled to resin using 5 equiv. 0.4 M 1-[Bis(dimethylamino)methylene]-1H-1,2,3-triazolo[4,5-*b*]pyridinium 3-oxide hexafluorophosphate (HATU) and 7.5 equiv. *N*,*N*-diisopropylethylamine (DIEA) for 20 min. Fmoc-*d*_8_-Val-OH was incorporated by manual SPPS using 1.5 equiv. amino acid, 1.5 equiv. HATU, and 3.3 equiv. DIEA. After each manual coupling, completion was assessed by qualitative ninhydrin test and the residue was recoupled in the advent of blue color. Peptides were cleaved from the resin and side chain deprotected using 10 mL of a standard cleavage cocktail of trifluoroacetic acid (TFA) containing 2.5% triisopropylsilane and 2.5% H_2_O (v/v) while agitating for 2 hours at room temperature. TFA was evaporated to 25% under N_2_ gas, peptide was precipitated using 35 mL chilled diethyl ether, and the precipitate was collected and purified by preparative HPLC (Waters Autopurify prep LC with diode array and QDa mass spec). Peptides were characterized by analytical LC/MS on a Waters Acquity I-Class UPLC with diode array and time of flight mass spec (Waters G2-XS) (ESI†).

### Steady-State FTIR Spectroscopy

Pure labeled or unlabeled peptides were massed, dissolved in diH_2_O (Milli-Q, resistivity 18.2 MΩ*cm), aliquoted into fractions, lyophilized, and then re-dissolved in diH_2_O at a concentration of 8 mM. Immediately prior to loading into the IR cell, peptide stock solution was diluted 1 : 1 with folding buffer (100 mM bis[tris(hydroxymethyl)methylamino]propane (BTP), 300 mM NaCl, pH 7.4 solution) at 4C. Approximately 10 μL of this solution was loaded at 4C into a demountable liquid cell (Harrick Scientific Products, Inc. model TFC-M13) with CaF_2_ window and a 75 μm teflon spacer. Samples were held at 4C for collection of ungelled condition and heated to 37C for 30 min to induce self-assembly using a circulating water bath with temperature control (Fisher Scientific Isotemp Refrigerated Circulator Model 9100). Spectra at 4 and 37C were collected in a Bruker Equinox 55 FTIR spectrometer with liquid N_2_ cooled MCT detector continuously purged with dry N_2._ 8000 spectra with a 2 cm^−1^ resolution were averaged. As described previously, the unlabeled MAX1 spectrum was auto-subtracted from all spectra in OPUS software. The resulting spectra were background corrected in MATLAB with a higher order polynomial function and fit with a pseudo-Voigt profile (ESI†) to determine the intensity, peak frequency, and full-width half-maximum (fwhm).^13*c*^

### Time-Resolved FTIR Spectroscopy

For time-resolved IR experiments, a four-syringe stopped-flow mixer (SFM 4000, BioLogic) equipped with an umbilical link to a Ball-Berger mixer was connected to a demountable liquid cell (CaF_2_ window, 75 μm pathlength). The umbilical link consists of two 45 cm long flow lines, each requiring 200 μL of liquid to fill the line. The dead time of the stopped-flow mixing was less than 100 ms. Approximately 700 μL of 8 mM peptide in diH_2_O was loaded into syringe 1, the folding buffer (100 mM BTP, varying amounts of NaCl, pH 7.4) was loaded into syringe 2, and the remaining two syringes were filled with diH_2_O for washing. Using the rapid-scan mode of the Bruker Equinox 55 instrument, 3 scans/sec were acquired at a spectral resolution of 2 cm^−1^. The average of 15 single scans was recorded, resulting in an effective time resolution of 5 s over a total of 1056 time points, or approximately 85 min. Initially, the cell was filled with folding buffer, and after acquiring 300 scans for background signal, 63 μL each of peptide solution and folding buffer were mixed and injected into the cell. Following data collection, the cell was rinsed with at least 2 mL of diH_2_O and equilibrated with 500 μL of folding buffer to prepare for the next replicate. Using Python packages NumPy and Pandas, the time-resolved series of spectra were background subtracted, corrected with a higher order polynomial function to smooth the baseline, and the resulting spectra were averaged with a sliding window of 10 time points, or 49.3 s. To find the peak position for each spectrum over time, each was fit to a single Gaussian function, using the Python package LMFIT. The time-dependent change in peak frequency was fit to a single exponential function for the time constant of the self-assembly process.

### Time-resolved circular dichroism

The conditions used for time-resolved circular dichroism were intended to closely replicate those used for time-resolved FTIR. To increase the signal to noise ratio in the far UV from a relatively high concentration of MAX1 peptide solution containing high salt, a 0.1 mm path length demountable UV quartz cuvette was used for circular dichroism measurements. Immediately prior to loading the cuvette, peptide stock solution (8 mM MAX1) was diluted 1 : 1 with folding buffer (100 mM BTP, 500 mM NaCl, pH 7.4) and maintained at 4C. Once the sample was prepared and loaded into the cuvette, it was quickly inserted into the circular dichroism instrument (Jasco J-1500) which was held at 23C, and data collection at 220 nm with 0.1 s time resolution was initiated.

## Results & discussion

The MAX1 β-strand hairpin is comprised of alternating valine and lysine residues, resulting in a hydrophobic and a hydrophilic face of each hairpin. To obtain site-specific self-assembly kinetics from distinct regions of the MAX1 peptide, variants of MAX1 were chemically synthesized using Fmoc SPPS with perdeuterated valine individually distributed along the strands of the hairpin peptide (Scheme 1). Perdeuterated valine, (*d*_8_)Val, was chosen over perdeuterated lysine because the asymmetric stretches of deuterated methyl groups of valine were previously shown to produce more intense and narrower IR absorption compared to deuterated methylenes of lysine.^19^ In addition, since lysine residues are on the hydrophilic face, the gel transition may produce smaller changes in the absorption spectra. Sufficient resolution between the initial and final states is an important requirement for time-resolved IR experiments where spectral and temporal resolution are at odds. To probe distinct environments within the peptide, (*d*_8_)Val was individually installed at Val1 and Val20 which are terminal, Val5 and Val16 which are located in the middle of the two β-strands, and Val9 which is located at the beginning of the type II' β-turn (Scheme 1). To confirm that incorporation of (*d*_8_)Val is indeed nonperturbative to MAX1, we compared the viscoelastic behavior of MAX1 to (*d*_8_)Val5 labeled MAX1. As expected, the rate of gelation and ultimate storage and loss moduli were similar for both peptide gels within experimental error (ESI Fig. S6†)

### Spectral analysis of C–D stretch absorptions

**Fig. 1:**
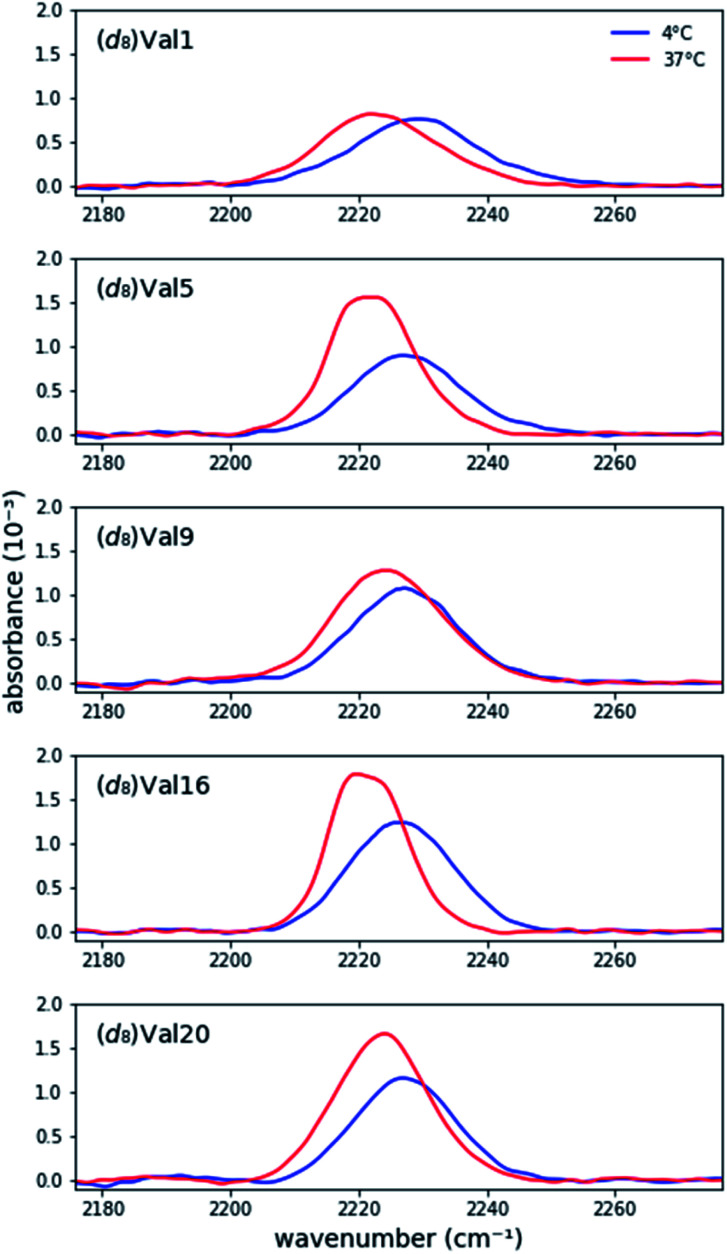
Background-corrected asymmetric stretch absorptions of the five individually (*d*_8_)Val-labeled MAX1 peptides. The peptide is unfolded at 4C and forms a gel at 37C.

Prior research has shown that MAX hydrogels do not form hydrogels at 4C, and the peptide remains in a random coil conformation.^4*e*,7^Fig. 1 shows asymmetric stretch absorptions of (*d*_8_)Val labeled peptides (4 mM in folding buffer) at 4C, where the peptide is unfolded, and 37C, where the peptide is folded. These unfolded spectra were virtuallly identical to those of peptide in water at room temperature, confirming that the peptide was indeed in its unfolded state. (ESI Table S1†). For each residue, the unfolded spectrum at 4C was well fit to a single pseudo-Voigt function with peak frequency of ∼2227 cm^−1^ and line width of ∼20 cm^−1^ (Table 1) with primary Gaussian contribution (*m* < 0.5, ESI, Fitting and analysis†), consistent with prior characterization of C–D bonds.^13*c*^ The predominant Gaussian line shapes imply that inhomogeneous broadening is the dominant broadening mechanism. Since two pairs of asymmetric stretches from the (*d*_3_)-methyls of valine are expected to contribute to the signal, observation of a single absorption implies that the stretches are degenerate, and the side chains experience a homogeneous electric field in the unfolded state. Moreover, since site-specific differences between the peak frequencies and line widths are minimal, each site experiences a similar local environment, indicating that the peptide adopts highly unstructured and solvated backbone conformations in the unfolded state. Absorptions from the symmetric stretches of (*d*_3_)-methyls, C_α_–D, and C_β_–D, are too weak to be detected.^20^

**Table 1:** Spectral fit parameters

	*T* (°C)	Absorbance (10^−3^)	Peak Frequency (cm^−1^)	Width^a^ (cm^−1^)
(*d*_8_)Val1	4	0.8	2228.6 ± 0.3	23.4 ± 0.6
	37	0.8	2223.2 ± 0.6	21.8 ± 1.4
(*d*_8_)Val5	4	0.9	2227.5 ± 0.2	19.8 ± 0.8
	37	1.6	2222.0 ± 0.1	15.4 ± 0.8
(*d*_8_)Val9	4	1.1	2227.3 ± 0.3	19.2 ± 0.9
	37	1.3	2224.6 ± 0.3	20.0 ± 0.5
(*d*_8_)Val16	4	1.3	2226.7 ± 0.1	18.8 ± 0.8
	37	1.9	2221.4 ± 0.3	13.8 ± 0.7
(*d*_8_)Val20	4	1.1	2228.0 ± 0.4	17.4 ± 0.4
	37	1.6	2223.7 ± 0.4	17.1 ± 0.5

a Width denotes the full width at half maximum absorbance (fwhm).

Each labeled MAX1 peptide sample underwent temperature-induced gelation for 30 min at 37C, and for each labeled position a red shift was observed in the C–D bond absorption band. Red shifts of up to 8.0 cm^−1^ in the IR stretch frequency of C–D bonds, corresponding to changes in dielectric constant ranging from 78.4 to 2.4, have been observed.^17^ The observed red shift (Fig. 1 and Table 1) is consistent with a change from a hydrophilic local environment in the unfolded state to a hydrophobic local environment in the interior of the assembled fibrils.

Considering the similarities of side chain positions along the peptide backbone, spectral features can be placed into three groups: terminal (Val1 and Val20), middle (Val5 and Val16), and turn (Val9) residues. For N- and C- terminal residues Val1 and Val20, the absorption peak frequencies red-shift by 5.4 cm^−1^ and 4.3 cm^−1^, respectively, relative to the unfolded state; however, the changes in line widths are marginal. This similarity in line width between solution and gel suggests that the terminal valine side chains retain a similar level of conformational dynamics, suggesting that the packing of these residues in the core of the fibril is not tight. However, these side chains become less solvated in the gel, consistent with their transfer to the more hydrophobic environment of the fibril.

For Val residues in the middle of the β-strand, the absorption peak frequencies are red-shifted by 5.5 cm^−1^ for (*d*_8_)Val5 and 5.3 cm^−1^ for (*d*_8_)Val16, exhibiting the lowest peak frequency values of the five labels. Interestingly, the line widths become significantly narrower upon adoption of the structured form. The widths (fwhm) of (*d*_8_)Val5 and (*d*_8_)Val16 are 15.4 cm^−1^ and 13.8 cm^−1^, respectively, which are narrower by 4.4 cm^−1^ and 5.0 cm^−1^ relative to the unfolded state (Table 1). As line width narrowing is consistent with the signal enhancement, it indicates that the motions of these valine side chains are much more restricted in the gelled state (Fig. 1). This observation is consistent with the expectation that gelation results in increased structural rigidity. However, rigidification appears to only be occurring in the hydrophobic interior of the folded peptide and is not observed in terminal or turn positions, at the edges of the fibril.

Upon transition to gel, residue Val9, positioned at the beginning of the hairpin turn and adjacent to the more polar Thr residue, produces an intermediate red shift of 2.7 cm^−1^ with virtually no change in line width. These measurements are consistent with the location of Val9 methyl groups on the edge of the hydrophobic core where they are in a partially desolvated and not fully packed environment.

Overall, the steady-state FTIR data indicate that although all five valine side chains are buried in the hydrophobic layer of gelled MAX1 amphiphilic fibrils, differences in the electrostatic environment along the hairpin are apparent. Notably, the red shift is smallest for Type II' β-turn residue Val9, somewhat larger for the terminal Val1 and Val20, and largest for the core residues Val5 and Val16. These results suggest that there is localized variation in solvation inside the hydrogel. In addition, a significant rigidification is only observed for the core residues, again suggesting that there is a tightly packed, desolvated core. These insights demonstrate the utility of our approach to provide complementary information to the previously reported solid-state NMR experiments.^5*b*^ It was previously shown that MAX hydrogels are made up of kinetically trapped 3.5 nm wide and 2.7 nm tall monomorphic fibrils consisting of β-strand hairpins aligned *syn* within a β-sheet but *anti* between layers (Scheme 1A and C).^5*b*^ The present study demonstrates that the hydrophobic environment created between these layers is more rigid toward the center (Val5, 16). Tycko and Schneider postulated that the *anti* alignment of hairpins across the β-sheet layers packs the N- and C-terminal valines into the diproline turn residues of its bilayer partner. Consistent with this model, we find that both the terminal residues and V9, located next to the diproline turn, remain more solvated than the inner packed residues.

### Comparison of enantiopure and racemic MAX

**Fig. 2:**
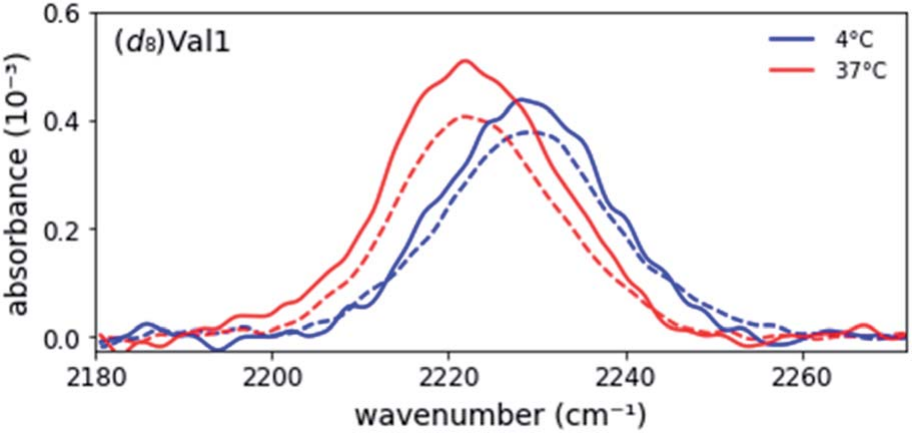
Representative overlay of asymmetric stretch absorption of (*d*_8_)Val1-labeled enantiopure (−) and racemic (−) MAX1 peptide in the unfolded (at 4 °C) and gel (at 37 °C) states. Enantiopure data is scaled by ½ to account for dilution of signal by addition of unlabeled D-MAX1.

Previously, it has been shown that a racemic MAX1 gel (equimolar mixture of enantiomers) is more rigid than either enantiopure gel, resulting from the formation of heterochiral fibrils that pack to form a more compact arrangement of valine residues in the hydrophobic core.^8,21^ To probe differences in the hydrophobic core of these two fibrils, IR spectra of racemic gels were collected, *i.e.*, equimolar mixtures of (*d*_8_)Val labeled MAX1 and unlabeled DMAX1 peptides. The absorption spectra of all five labeled positions in the racemic gels overlapped nearly perfectly with the spectra of the enantiopure gel (Fig. 2 and ESI Table S2, Fig. S1†). As the C–D stretch frequencies and line widths are very sensitive to polarity of their microenvironments, the data indicate that not only are the hydrophobic environments in both types of fibrils highly similar, but the side chains are similarly flexible. Nonetheless, the racemic MAX1 gel possesses unique characteristics, including a four-fold increase in stiffness in comparison to the enantiopure gel. In our study, the labeled valine residues probe the exterior (Val1, Val9, and Val20) and the most rigid part of the core (Val5 and Val16), regions that are highly similar in the racemic and enantiopure gels.

### Time-resolved studies

SF-FTIR spectroscopy is well suited to follow kinetics in the presence of phase transitions because the signal measures the effect of the local environment on bond vibrations of the probe and FTIR is not impeded by solubility or phase changes. In the case of MAX1 hydrogels, a relatively high concentration (4 mM) both promotes desired materials properties of the hydrogel and provides high signal to noise in the IR absorption spectra. MAX1 (*d*_8_)Val labeled peptides with absorption frequency shifts greater than 4 cm^−1^ between the gelled and un-gelled form in the steady-state were selected for time-resolved experiments ((*d*_8_)Val1, (*d*_8_)Val5, (*d*_8_)Val16, and (*d*_8_)Val20). Due to instrumental limitations, SF-FTIR experiments were required to be performed at room temperature. However, at room temperature and under the buffer conditions used in the steady-state experiments (50 mM BTP pH 7.4, 150 mM NaCl), self-assembly, as judged by the red shift of the C–D stretch vibrations, does not occur on a reasonable time scale (ESI Fig. S2A†). Interestingly, gelation of MAX1 has previously been shown to be inducible at room temperature using a high salt-content buffer.^4*b*^ We confirmed that increasing the salt concentration accelerates self-assembly (ESI Fig. S2, Table S3†), and used 250 mM NaCl in 50 mM BTP, pH 7.4, for subsequent experiments. The completion of fibril formation was further confirmed by circular dichroism (ESI Fig. S3†). Importantly, rheology shows that gelation is facilitated at these experimental conditions (ESI FS6†).

**Fig. 3:**
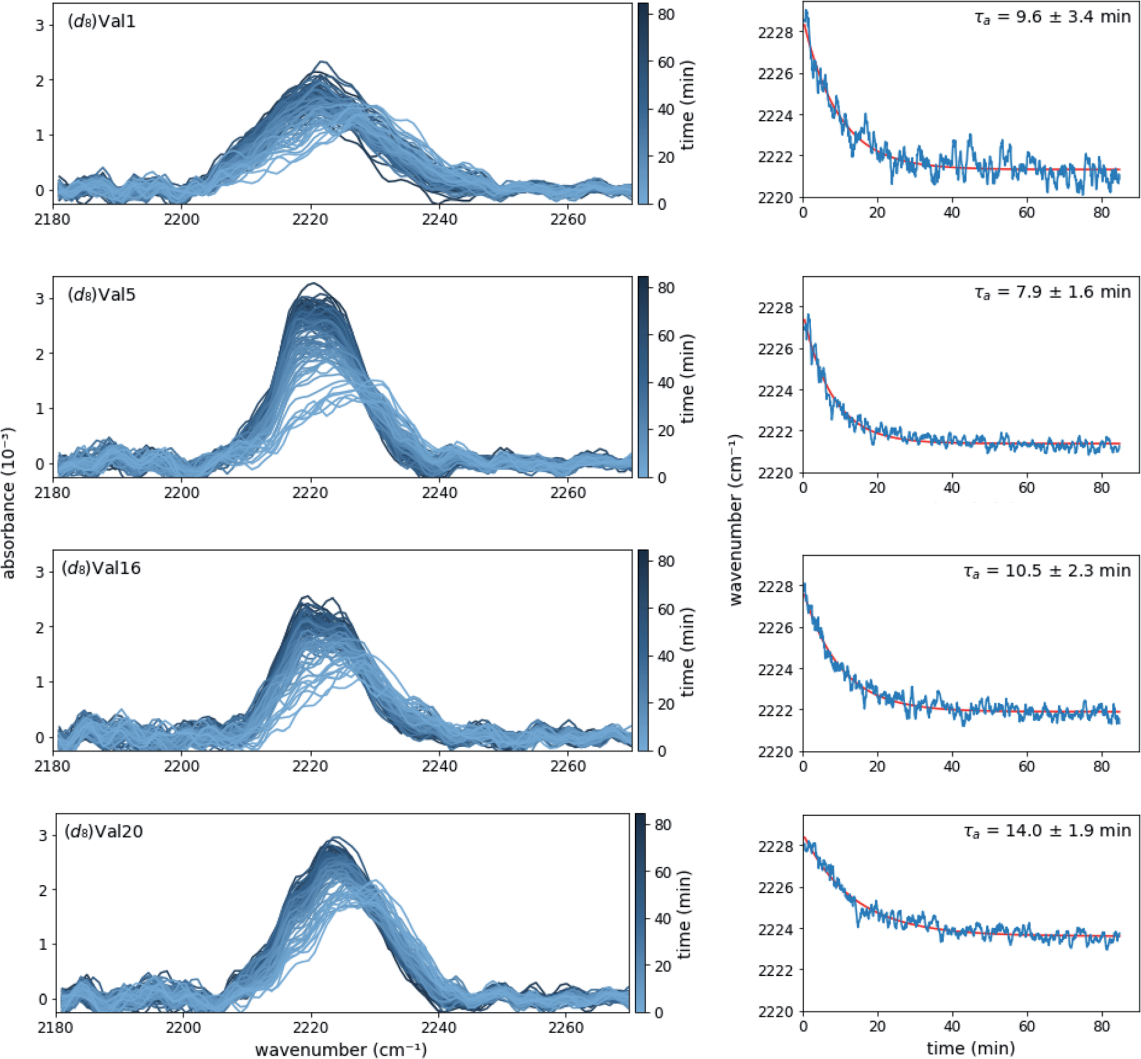
Absorption spectra (left panels) and the corresponding peak frequencies as a function of time (right panels) of (*d*_8_)Val-labeled MAX1 peptides after mixing equal volumes of 8 mM MAX1 in water with 100 mM BTP pH 7.4, 500 mM NaCl. Exponential fits to the time traces in the right panels are shown in red. Each spectrum and trace are the average of at least three data sets.

Time-dependent absorption spectra and peak frequencies are shown in Fig. 3. The change in peak frequency fit well to a single-exponential functions for all four labelling positions investigated, yielding site-specific assembly time *τ*_a_. As one may expect, the assembly time for the terminal residues (Val1 and Val20) are identical within experimental error, as are the assembly times for the core residues (Val5 and Val16). When averaged, the assembly times of these core residues is somewhat faster (9.6 ± 3.4 min) than the assembly time of the terminal residues (11.8 ± 2.8 min), but given the experimental error, the difference is not significant. The similarity in rates between different residues in the peptide suggests that with respect to each site the self-assembly process is cooperative in nature. In addition, although lag phases of up to several minutes have been observed for self-assembly processes, our data does not reveal a lag phase for the valine desolvation transition on the time scale used in the FT-IR study.^15*c*^

While rheology supports the evolution of viscoelasticity on the time scale of the peak shifts observed by FTIR (time constant ∼10 min), significant increases to the rigidity of the gel continue for over 3 hours (ESI Fig. S6A†). This suggests that the hydrophobic packing of the core, likely correlated to fibril formation, as measured by FTIR, is a distinct process from the maturation of the gel, as measured by rheology. The site-specific FTIR method reports precisely on the local environment of an incorporated probe, whereas rheology measures the viscoelasticity, a bulk mechanical property that is reliant on a number of other physical transitions.

For comparison, we also performed time-resolved circular dichroism experiments of unlabeled MAX1 peptide (ESI Fig. S4†). To control for any differences between the unlabeled MAX1 and the deuterated species, these experiments were repeated with (*d*_8_)Val5 labeled MAX1 and time constants, exponential fit, and the steady state spectrum were found to be within error of the unlabeled peptide (ESI Fig. S5†). When fit to a single-exponential function, the time for evolution of β-sheet structures observed *via* circular dichroism, 10.6 ± 0.3 min, is in good agreement with the *τ*_a_ observed in the SF-FTIR experiments (Fig. 3). The similarity of time constants for circular dichroism, which measures the β-strand structure of the peptide, and site-specific SF-FTIR, which measures the formation of the hydrophobic core, implies that these physical processes are concerted.

However, the circular dichroism data is best fit with two exponentials, yielding time constants of 4 min (60% amplitude) and 21 min (40% amplitude) (ESI Fig. S4†). Due to the larger error bars, we cannot rule out that this multiexponential behavior is also present in the SF-FTIR kinetics. However, the SF-FTIR experiments clearly show that the two exponentials are not due to local differences in self-assembly, since the time constants for core and terminal residues do not correlate with the two circular dichroism derived components. This demonstrates the advantage of using multiple techniques to elucidate different aspects and to fully characterize the self-assembly kinetics. Since the C–D stretch absorptions observed in SF-FTIR report on the local environment of the valine side chains, which are buried in the hydrophobic core of the MAX1 hydrogel, the frequency shifts observed by SF-FTIR report on the formation of this hydrophobic core between two β-hairpins. In contrast, circular dichroism measures the global average of the MAX1 peptide backbone conformation, *i.e.* the transition from random coil to β-hairpin. The differences between the SF-FTIR and circular dichroism kinetics could be explained by multiple, distinct assembly processes taking place during the gelation of MAX1, as has been suggested for other hydrogels, including peptide hydrogels.^22^ Future experiments, such as determining the self-assembly kinetics of additional residues *via* SF-FTIR could provide a more complete picture of the hydrogelation of this promising peptide hydrogel.

## Conclusions

The site-specific incorporation of C–D bonds has been established for the direct observation of bond vibrations in peptides and proteins. Here we extend these studies to the realm of kinetic analysis through combining SF-FTIR spectroscopy with site-specific C–D labeled MAX1 peptides. The data shown herein demonstrate that site-specific kinetics can be reliably followed using C–D bonds as an IR probe of self-assembly of MAX when stopped-flow is coupled with a rapid-scan FTIR spectrometer. Observation of distinctive regions of the hydrophobic core of MAX1 fibrils supports side chain rigidification in the middle of the β-strands. FTIR monitoring of distinct sites yields similar time constants for residues located in the core and at terminal ends of the peptide, consistent with cooperative assembly. Comparison with non-site-specific methods such as circular dichroism and materials measurements such as rheology emphasizes the utility of site-specific SF-FTIR for precisely characterizing molecular level transitions that can be distinct from macroscopic properties. These results suggest that the coupling of SF-FTIR with a variety of site-specific nonperturbative probes holds promise for the time-resolved characterization of protein function such as folding and self-assembly, even in larger and more complex systems.

## Data availability

The data that support these findings are available in the ESI† and from the corresponding author upon reasonable request.

## Author contributions

Z. C. A.: methodology, software, validation, formal analysis, investigation, data curation, writing—original draft, writing—review & editing; E. J. O.: methodology, investigation, writing—original Draft; T. L. L.: investigation, writing—review & editing; Z. L.: resources; A. Y. K.: software; M. H.: software; J. Z.: methodology, writing—review & editing, supervision; R. A.: methodology, writing—original draft, writing—review & editing, supervision; P. E. D.: conceptualization, resources, writing—review & editing, supervision.

## Conflicts of interest

There are no conflicts to declare.

## Supplementary Material

SC-013-D1SC06562A-s001
