# Supplementary material for: Direct observation of peptide hydrogel self-assembly
Source: Chem Sci. 2022 Aug 16;13(34):10020–8. doi: 10.1039/d1sc06562a (PMC9430618; doi:10.1039/d1sc06562a)
Supplement: SC-013-D1SC06562A-s001 [file SC-013-D1SC06562A-s001.pdf]

Electronic Supplementary Material (ESI) for Chemical Science.
This journal is © The Royal Society of Chemistry 2022

Supporting Information

Direct Observation of Peptide Hydrogel Self-Assembly

Zoë C. Adams, Erika J. Olson, Tania L. Lopez-Silva, Zhengwen Lian, Audrey Y. Kim, Matthew Holcomb, Jörg Zimmermann, Ramkrishna Adhikary, Philip E. Dawson

Table of Contents

Materials and Methods *2*

Materials 2

Analysis and Purification 2

Steady-State Buffer vs. Water Control Experiment 2

Buffer Optimization 3

Oscillatory Rheology 3

Fitting and Analysis 3

FTIR Peak Visualizer 4

Table S1: Spectral Parameters for MAX1 in water and buffer *5*

Table S2: Spectral parameters for racemic gels, consisting of 1:1 MAX1:D-MAX1 .... *5*

Fig. S1 Comparison of racemic and enantiopure MAX1 gels *6*

Fig. S2 Buffer Screen *7*

Table S3: Steady state statistics for all four positions used in the time-resolved studies in 250 mM NaCl gelling buffer *8*

Fig. S3 Circular dichroism of different buffers *9*

Fig. S4 Time-resolved circular dichroism *10*

Fig. S5 Circular dichroism of unlabeled vs. labeled MAX1 .... *10*

Fig. S6 Oscillatory rheology of unlabeled vs. labeled MAX1 .... *11*

Analytical HPLC Mass Spectrometry of Peptides (Fig. S7-S13) *12*

Materials and Methods

Materials

All reagents were obtained directly from commercial suppliers and used without further purification. Dimethylformamide, Dichloromethane, trifluoroacetic acid (TFA), and N,N-diisopropylethylamine from Sigma-Aldrich; acetonitrile (MeCN), diethyl ether, 4-methylpiperidine, 1,3-Bis[tris(hydroxymethyl)methylamino]propane (BTP), and sodium chloride from Fisher Scientific; HATU (1-[Bis(dimethylamino)methylene]-1H-1,2,3-triazolo[4,5-b]pyridinium 3-oxide hexafluorophosphate) from Oakwood Products Inc.; Fmoc-Lys(Boc)-OH, Fmoc-Val-OH, Fmoc-Pro-OH and Fmoc-Thr(tBu)-OH from Bachem; Fmoc-D-Pro-OH from Matrix Scientific; Fmoc-*d8*-Val-OH from CDN Isotopes; Rink-Amide Tenta Gel XV resin (loading capacity 0.2-0.4 mmol/g) from Rapp Polymere GmbH. Water was purified using a Millipore Milli-Q Q-Gard 2 water purification system to a resistivity of 18.2 MΩ*cm (diH_2O).

Analysis and Purification

Crude peptides were purified on preparative HPLC (Waters Autopurify prep LC with diode array and QDa mass spec). Peptides were characterized by analytical LC/MS on a Waters Acquity I-Class UPLC with diode array and time of flight mass spec (Waters G2-XS).

<u>Waters Preparative HPLC Mobile Phase and Gradient:</u>

Buffer A: H_2O (0.1% TFA)
Buffer B: MeCN

Initial:	10% B
1.0 minute:	10% B
9.0 minutes:	25% B
9.5 minutes:	95% B
12.0 minutes:	10% B

<u>Waters Analytical LC/MS Mobile Phase and Gradient:</u>

Buffer A: H_2O (0.1% Formic Acid)
Buffer B: MeCN

Initial:	5% B
0.4 minutes:	5% B
5.0 minutes:	99% B
5.4 minutes:	5% B

Steady-State Buffer vs. Water Control Experiment

FTIR spectra of 2 mM samples of each peptide were collected at room temperature. A 4 mM peptide stock in diH_2O was mixed 1:1 with either diH_2O (unfolded) or folding buffer (100 mM BTP, 300 mM NaCl, pH 7.4 solution) and immediately loaded into a demountable liquid cell with CaF_2 window and a 75 µm teflon spacer. The IR cell was then heated to 37 C and equilibrated for 30 minutes, followed by rapid cooling to 21 C prior to data collection of 8000 scans with a 2 cm^{-1} resolution. Results are averages of at least three independent experiments.

Buffer Optimization

To trigger gelation in the steady-state, 4 mM MAX1 peptide was dissolved in 50 mM BTP buffer, pH 7.4, containing 150 mM NaCl, and maintained at 4 C then incubated at 37 C for 30 min. SF-FTIR experiments were initially performed at room temperature under the buffer conditions in the steady-state experiments (4 mM MAX1 peptide in 50 mM BTP buffer, 150 mM NaCl, pH 7.4), by 1:1 mixing of 8 mM MAX1 peptide in H_2O with 2x folding buffer. However, the peptide was found not to achieve the desired gel properties on a reasonable time scale as determined by the red shift of the C-D stretch vibration (**Fig. S2A**). Interestingly, gelation of MAX1 has previously been induced at room temperature using high salt-content buffer.[1] We confirmed that increasing the salt concentration accelerates self-assembly; at 2.7x the original salt concentration gelation occurred too quickly to allow for sufficient time resolution (**Fig. S2B**). A salt concentration of 250 mM NaCl in 50 mM BTP (pH 7.4) afforded full fibril formation on a reasonable time scale (under 1 hr, room temperature) (**Table S3, Fig. S2**) The completion of the process was further confirmed by circular dichroism (**Fig. S3**).

Oscillatory Rheology

Rheological measurements were performed using a Discovery HR20 (TA Instruments) equipped with a 25 mm stainless steel parallel plate geometry and a 500 µm gap height. MAX1 and d5-MAX1 peptides were dissolved in cold diH2O (Milli-Q, resistivity 18.2 MΩ*cm) at a concentration of 8 ± 0.2 mM determined by UV-vis spectroscopy (ε_{220} 15,750 cm^{-1} M^{-1}). Peptide solutions were diluted 1:1 with ice-cold 2X BTP buffer (100 mM BTP, 500 mM NaCl, pH 7.4) immediately before rheology tests and placed on the cold rheometer stage at 5°C. Peptide solutions were equilibrated at 5°C for 1 min before increasing the temperature to 23°C at a rate of 17.45 °C/min. Samples were maintained at 23°C during the following rheological tests. Time-dependent viscoelastic behavior was analyzed at 0.2% strain and 6.0 rad/s for 3 h to monitor gel formation and material rigidity, followed by a frequency sweep test (0.2 % strain and angular frequency range from 0.1 to 100.0 rad/s) and an amplitude sweep test (oscillatory strain from 0.1 to 1000.0 % at 6.0 rad/s). Rheological data represent the mean G' and G'' obtained from four independent experiments. Standard deviation is represented as dotted lines.

Fitting and Analysis

The collected spectra were background corrected in MATLAB with a higher order polynomial function and fit with a pseudo-Voigt profile, I(ν):

$$I(\nu) = \frac{A}{m + \sqrt{\pi \ln 2}(1-m)}\left[m\frac{fwhm^2}{4(\nu-\nu_0)^2 + fwhm^2}\right] + \sqrt{\pi \ln 2}(1-m)\ exp\left(-\frac{4(\ln 2)(\nu-\nu_0)^2}{fwhm^2}\right)$$

where A is the amplitude at center frequency ν_0, *m* varies between 0 and 1 and describes the character of the pseudo-Voigt profile (which is purely Lorentzian for *m* = 1 and purely Gaussian for *m* = 0), and fwhm is the full width at half maximum of the pseudo-Voigt profile.

FTIR Peak Visualizer

For code used to analyze FTIR time-resolved and steady-state data, as well as user-friendly scripts for FTIR data analysis and peak plotting please visit:

https://github.com/dawsonlab/FTIR-PeakVisualizer

Table S1: Spectral Parameters for MAX1 in water and buffer

		Absorbance (10^{-3})	Peak Frequency (cm^{-1})	Width (cm^{-1})[a]
(d_8) Val1	Water	0.4	2231.1 ± 0.1	14.7 ± 0.8
	Buffer	0.8	2221.0 ± 0.2	20.4 ± 0.3
(d_8) Val5	Water	0.7	2227.3 ± 0.3	17.7 ± 1.4
	Buffer	1.1	2221.2 ± 0.4	14.6 ± 1.1
(d_8) Val9	Water	0.6	2227.8 ± 0.3	16.8 ± 0.5
	Buffer	0.8	2224.8 ± 0.4	19.2 ± 0.8

[a]Width denotes the full width at half maximum absorbance (fwhm).

Table S2: Spectral parameters for racemic gels, consisting of 1:1 MAX1:D-MAX1

	T (°C)	Absorbance (10^{-3})	Peak Frequency (cm^{-1})	Width (cm^{-1})[a]
(d_8) Val1	4	0.4	2228.8 ± 0.4	22.0 ± 1.6
	37	0.5	2222.9 ± 1.5	22.8 ± 1.1
(d_8) Val5	4	0.6	2227.5 ± 1.2	18.5 ± 1.0
	37	0.7	2222.8 ± 0.7	14.4 ± 0.5
(d_8) Val9	4	0.6	2227.1 ± 0.9	19.6 ± 1.1
	37	0.7	2224.8 ± 0.5	19.9 ± 1.7
(d_8) Val16	4	0.8	2226.5 ± 1.0	20.0 ± 1.7
	37	1.0	2221.6 ± 0.4	14.0 ± 1.8
(d_8) Val20	4	0.7	2227.6 ± 0.5	17.9 ± 1.7
	37	0.9	2224.3 ± 0.3	17.7 ± 2.6

[a]Width denotes the full width at half maximum absorbance (fwhm).

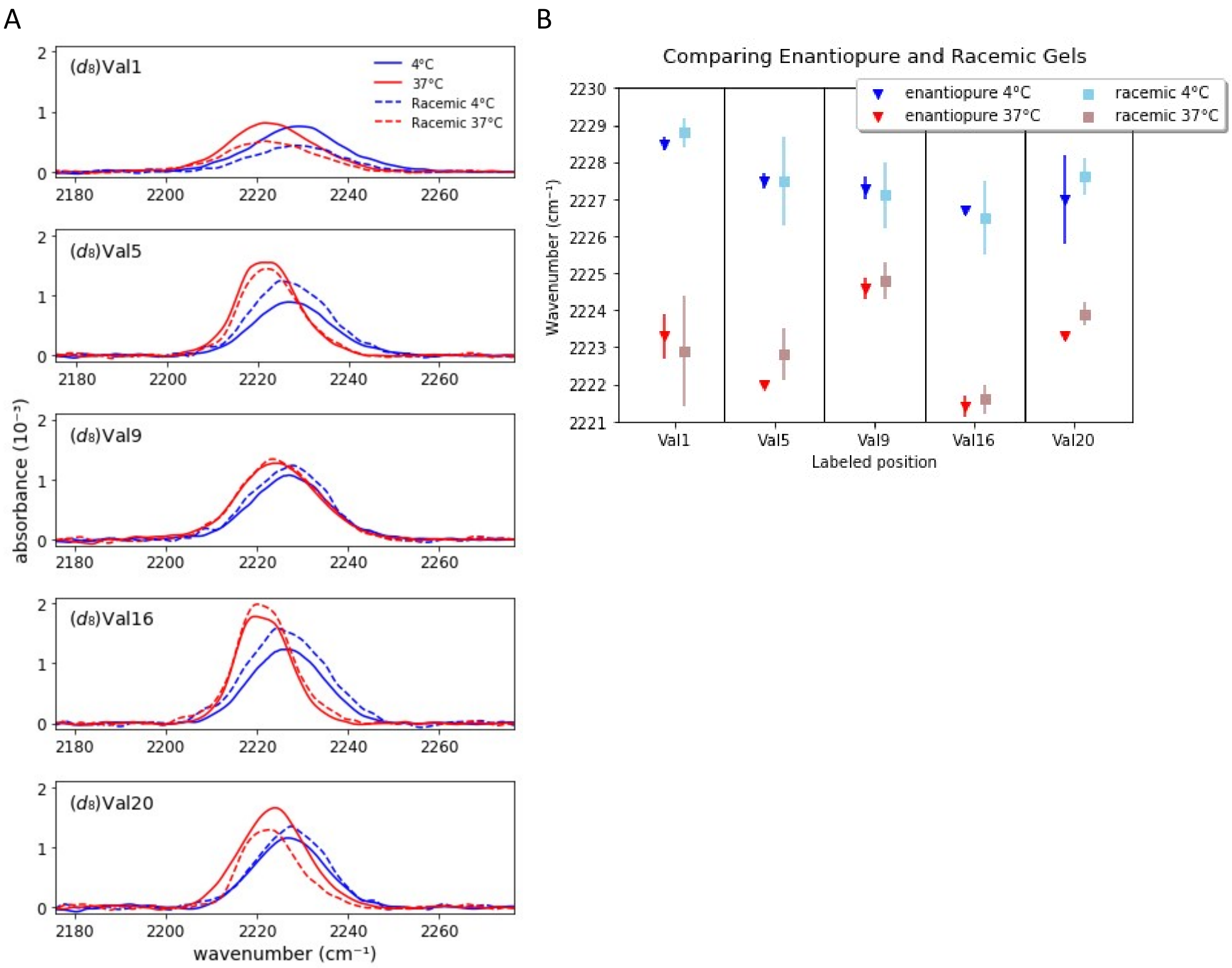

Fig. S1 Comparison of absorption spectra and peak frequencies for racemic and enantiopure labeled MAX1. (A) Asymmetric absorption bands of the (d_8)Val-labeled enantiopure and racemic MAX1 peptides. The peptide is unfolded at 4 °C and forms a gel at 37 °C. Dashed lines represent the IR absorption of the racemic gel, multiplied by two to account for loss of signal due to 1:1 dilution. (B) Comparison of peak frequencies for enantiopure and racemic MAX1 gels in the gelled and ungelled state.

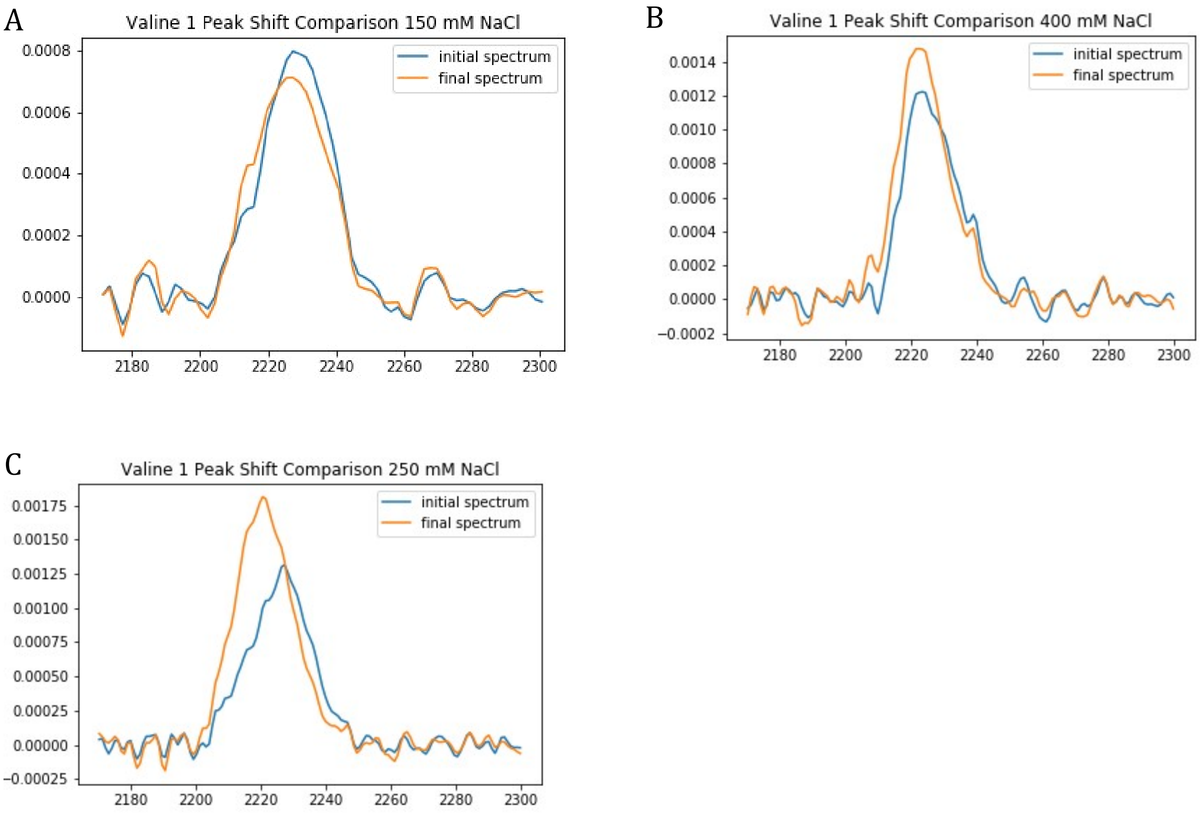

Fig. S2 Screen of buffer conditions to obtain a peak shift on a reasonable time scale for SF-FTIR (23 C) observation. A: 85 minutes after injection of peptide and gelling buffer with 150 mM NaCl, no discernable peak shift occurred. B: The earliest observable spectrum after injection of peptide and gelling buffer with 400 mM NaCl aligns with the final spectrum, indicating a very fast peak shift, which could not be resolved. C: A discernable peak shift was observed at room temperature after injection of peptide and gelling buffer with 250 mM NaCl. This condition was used for the time-resolved experiments.

Table S3: Steady state statistics for all four positions used in the time-resolved studies in 250 mM NaCl gelling buffer

	T (°C)	Absorbance (10^{-3})	Peak Frequency (cm^{-1})	Width (cm^{-1})[a]
(d_8) Val1	4	0.9	2228.6 ± 0.3	19.4 ± 1.0
	37	1.0	2224.1 ± 2.7	22.6 ± 1.3
(d_8) Val5	4	1.2	2227.4 ± 1.2	18.7 ± 0.8
	37	1.6	2223.3 ± 0.1	16.7 ± 0.2
(d_8) Val16	4	1.7	2224.9 ± 1.4	18.9 ± 1.0
	37	2.3	2222.2 ± 0.4	15.8 ± 0.3
(d_8) Val20	4	1.6	2225.2 ± 0.2	19.2 ± 1.6
	37	1.6	2224.3 ± 0.3	16.3 ± 1.2

[a]Width denotes the full width at half maximum absorbance (fwhm).

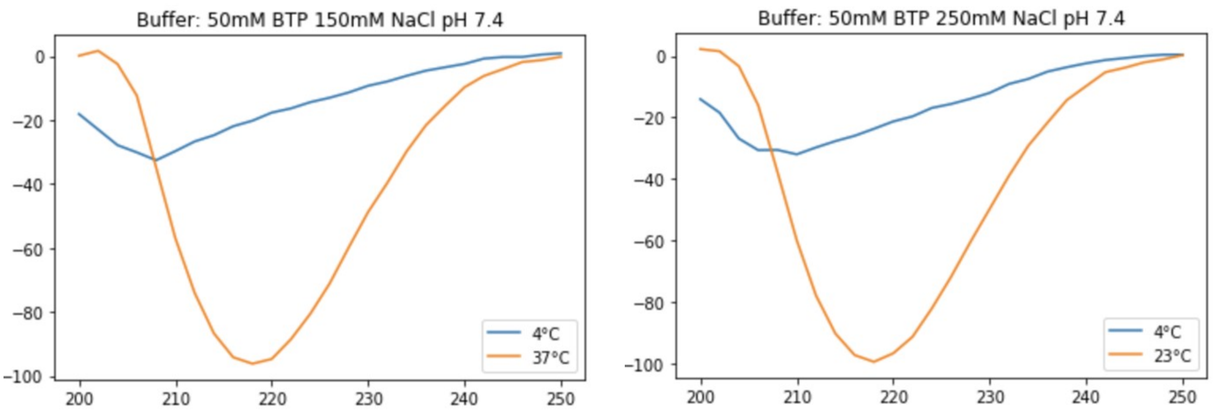

Fig. S3 Circular dichroism data for MAX1 in standard gelling buffer (50 mM BTP, 150 mM NaCl, pH 7.4) and high salt content gelling buffer (50 mM BTP, 250 mM NaCl, pH 7.4).

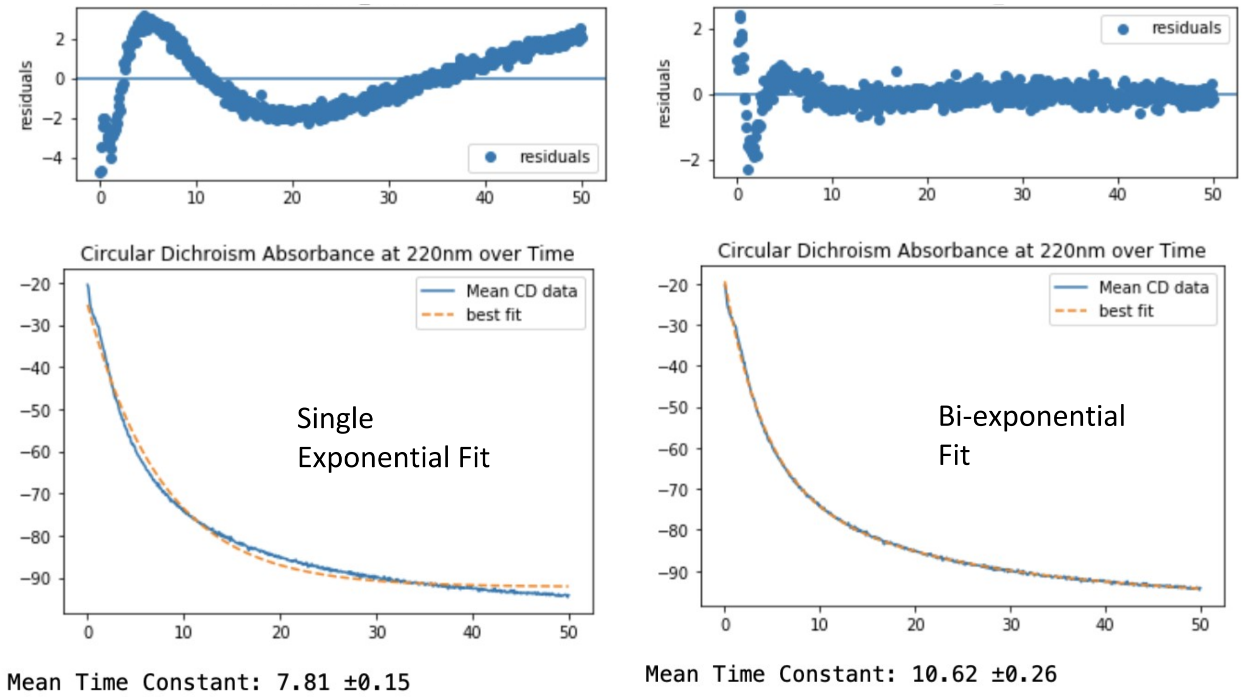

Fig. S4 Time-resolved circular dichroism data for unlabeled MAX1 in high salt content gelling buffer at 23 C. A two-term exponential is a superior fit for this data.

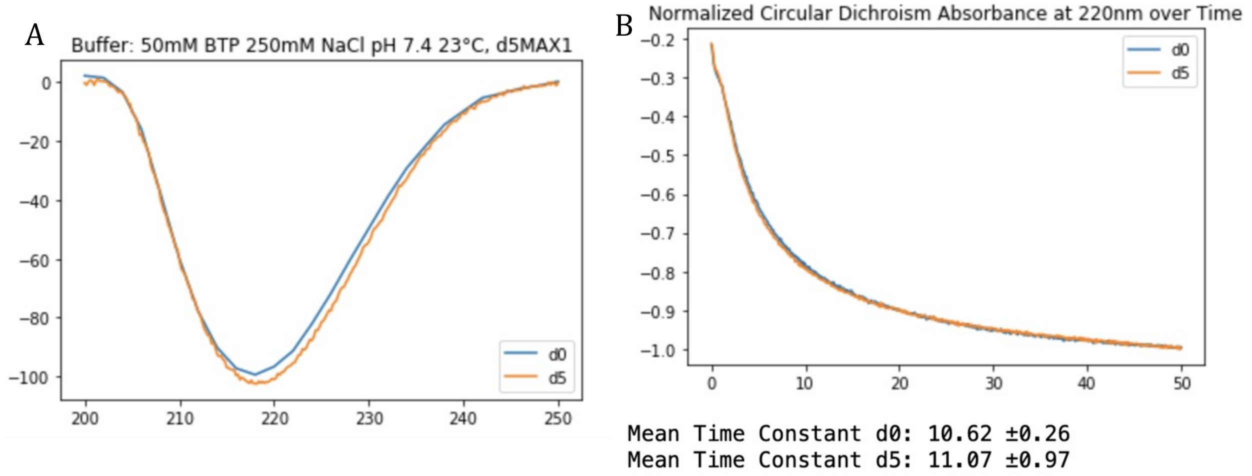

Fig. S5 Steady-state (A) and time-resolved (B) circular dichroism data comparing unlabeled MAX1 to (d_8)Val5 labeled MAX1 in high salt content gelling buffer at 23 C. A two-term exponential fit to each results in time constants within error.

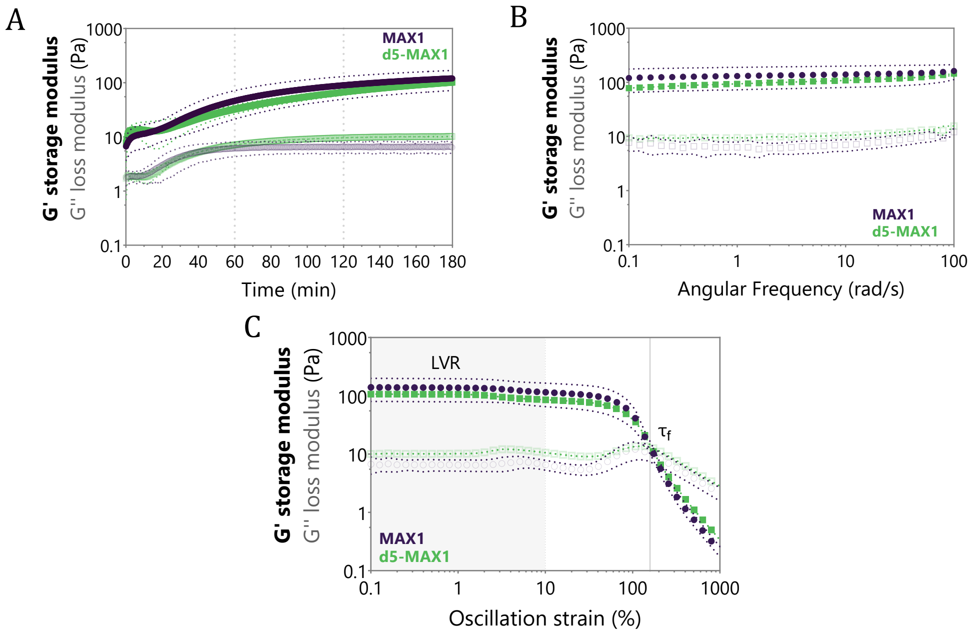

Fig. S6 (A) Time-Dependent viscoelastic behavior. Both peptides form hydrogels with storage moduli (G') of 119.8 ± 48.2 Pa for MAX1 and 100.8 ± 9.5 Pa for d5-MAX1 after 3 h at 23°C. These G' values are not significantly different (P value 0.498 by Welch's test). The loss moduli (G'') for MAX1 and d5-MAX1 were 6.4 ± 1.5 Pa and 10.1 ± 0.6 Pa, respectively. Both peptide solutions exhibited a gel character with G'>G'' at all times. However, G' increased over time which indicates the gel networks are evolving and becoming more rigid. (B) Frequency sweep. Both peptide gels are characterized by G' and G'' values that are linear over the frequency range tested. (C) Amplitude Sweep. Both peptide gels yield at similar strains indicating they form similar fiber networks. Finally, both strain and frequency sweep experiments indicate that the time-sweep experiment was performed in the linear viscoelastic region of both materials.

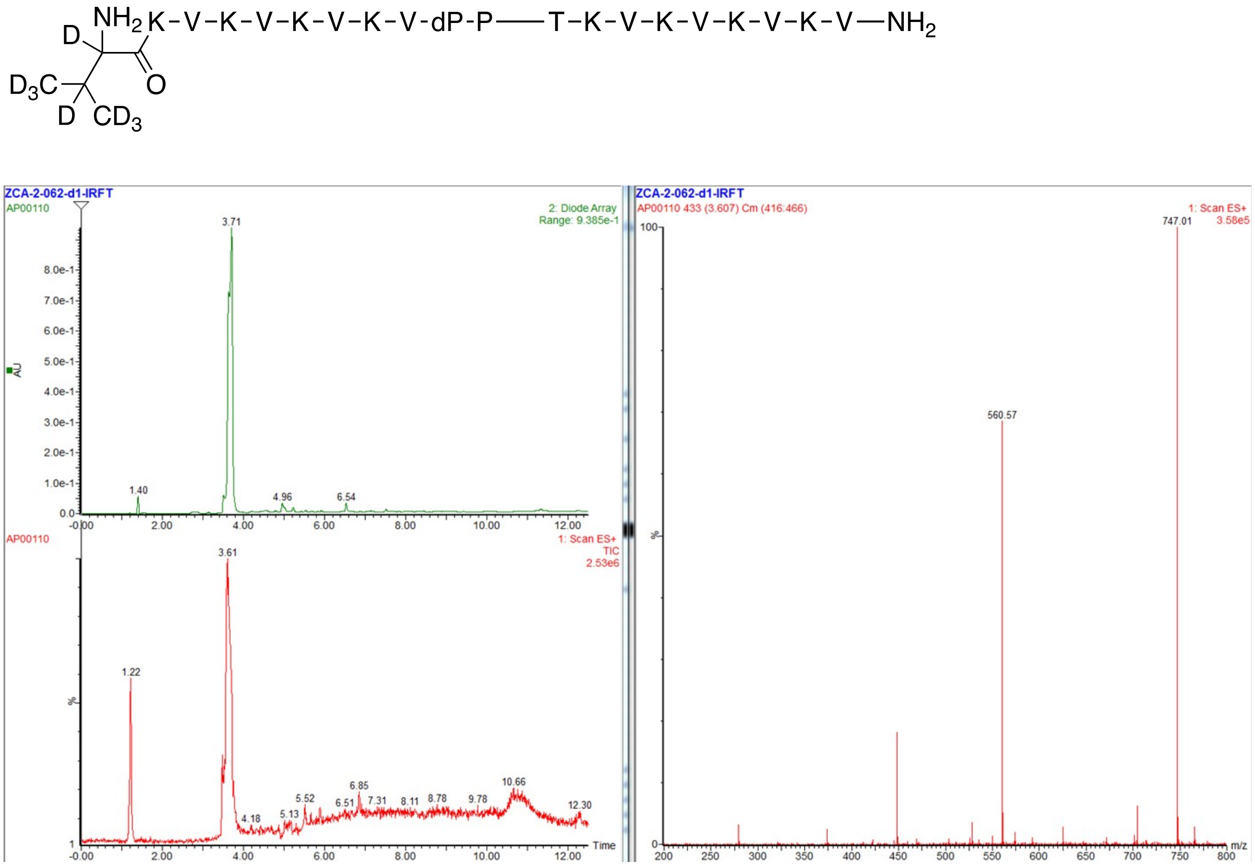

Fig. S7 LC/MS characterization of pure *(d₈)*-Val1 MAX1. Expected molecular weight 2238.0. Observed mass 2237.6. ~95% yield by UV

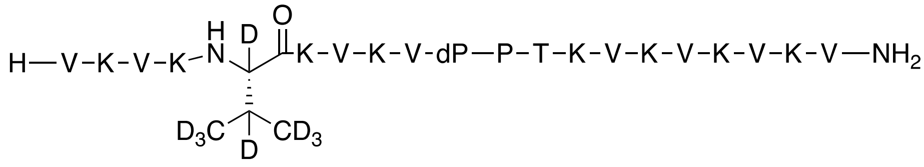

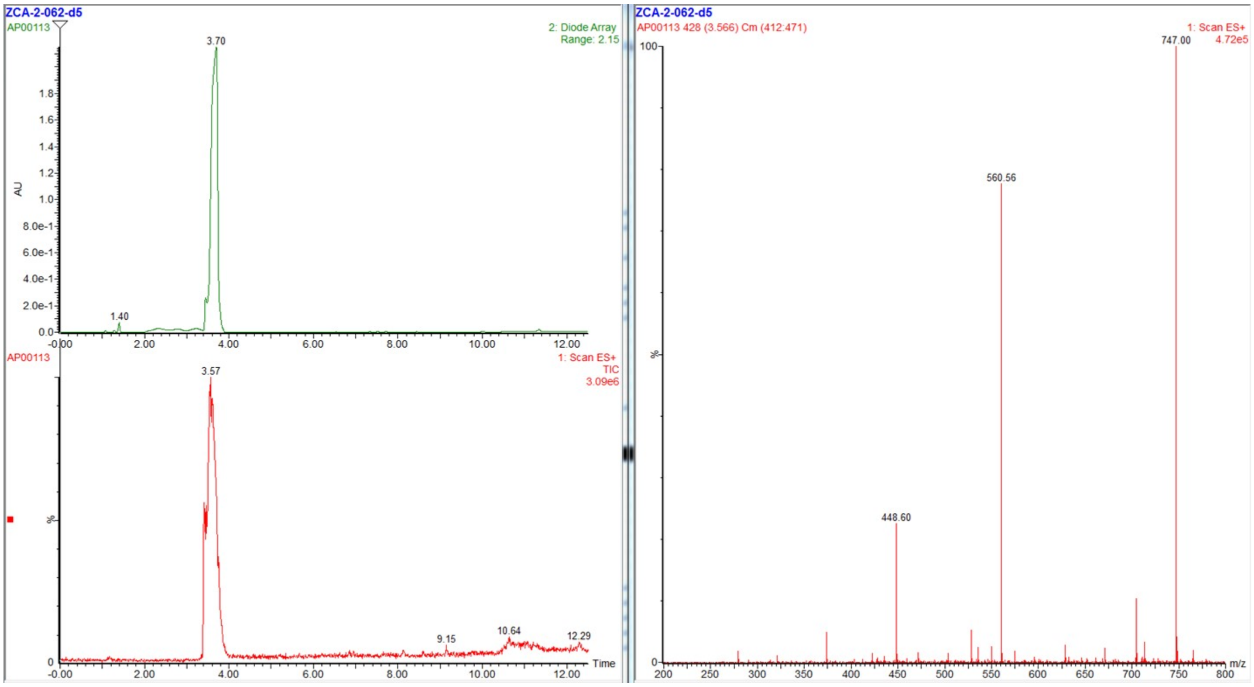

Fig. S8 LC/MS characterization of pure *(d_8)*-Val5 MAX1. Expected molecular weight 2238.0. Observed mass 2237.6. ~97% yield by UV

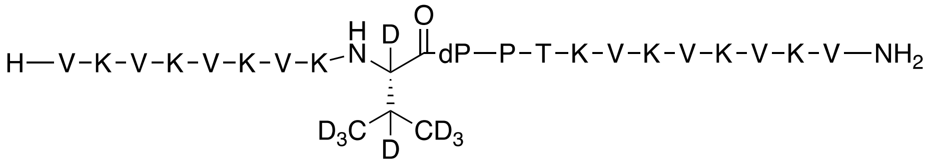

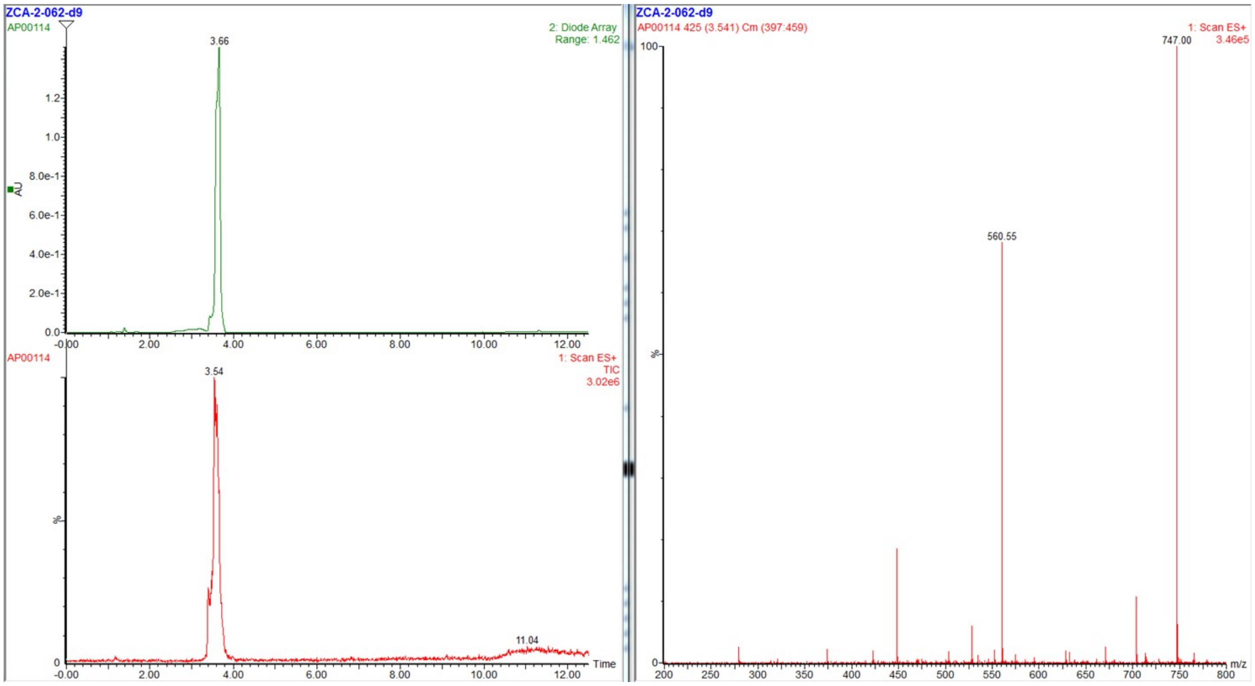

Fig. S9 LC/MS characterization of pure *(d_8)*-Val9 MAX1. Expected molecular weight 2238.0. Observed mass 2237.6. ~94% yield by UV

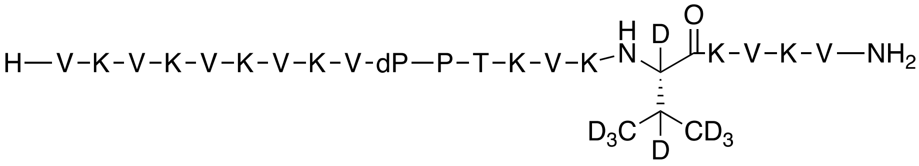

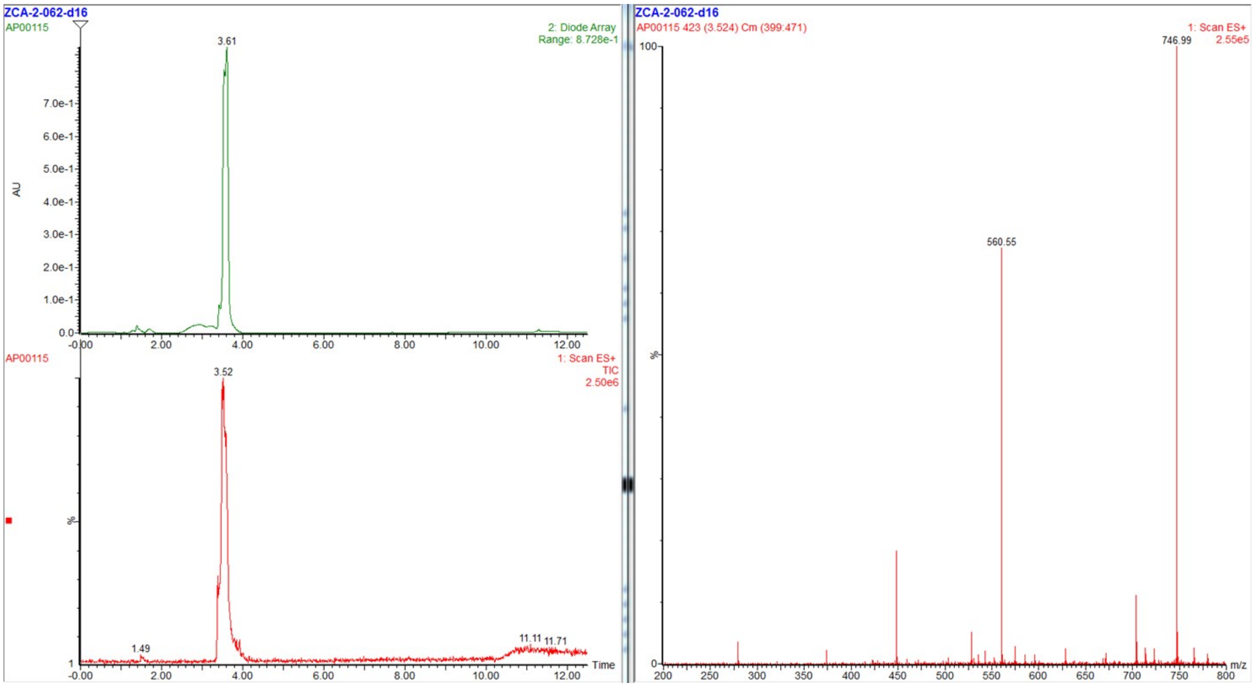

Fig. S10 LC/MS characterization of pure *(d_8)*-Val1 MAX16. Expected molecular weight 2238.0. Observed mass 2237.6. ~89% yield by UV

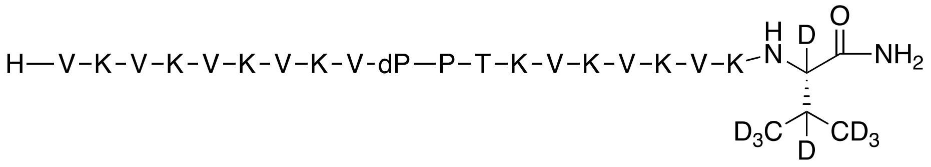

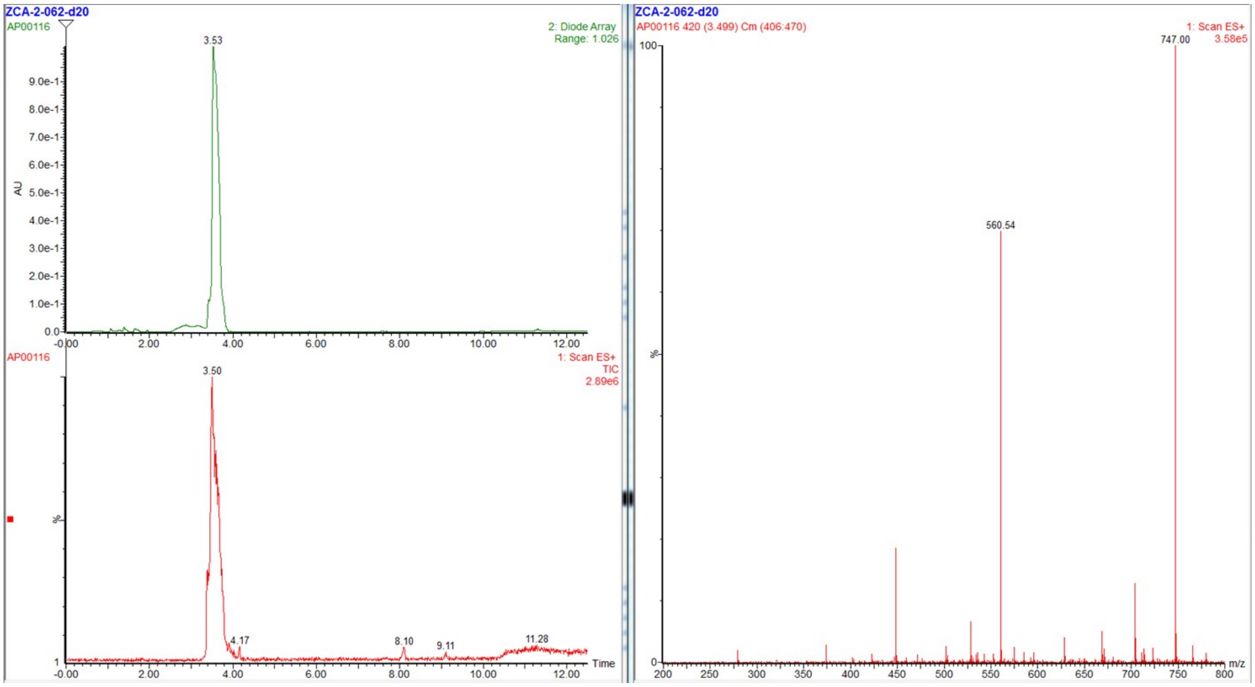

Fig. S11 LC/MS characterization of pure *(d_8)*-Val1 MAX20. Expected molecular weight 2238.0. Observed mass 2237.6. ~96% yield by UV

H—V-K-V-K-V-K-V-K-V-dP—P-T-K-V-K-V-K-V-K-V—NH_2

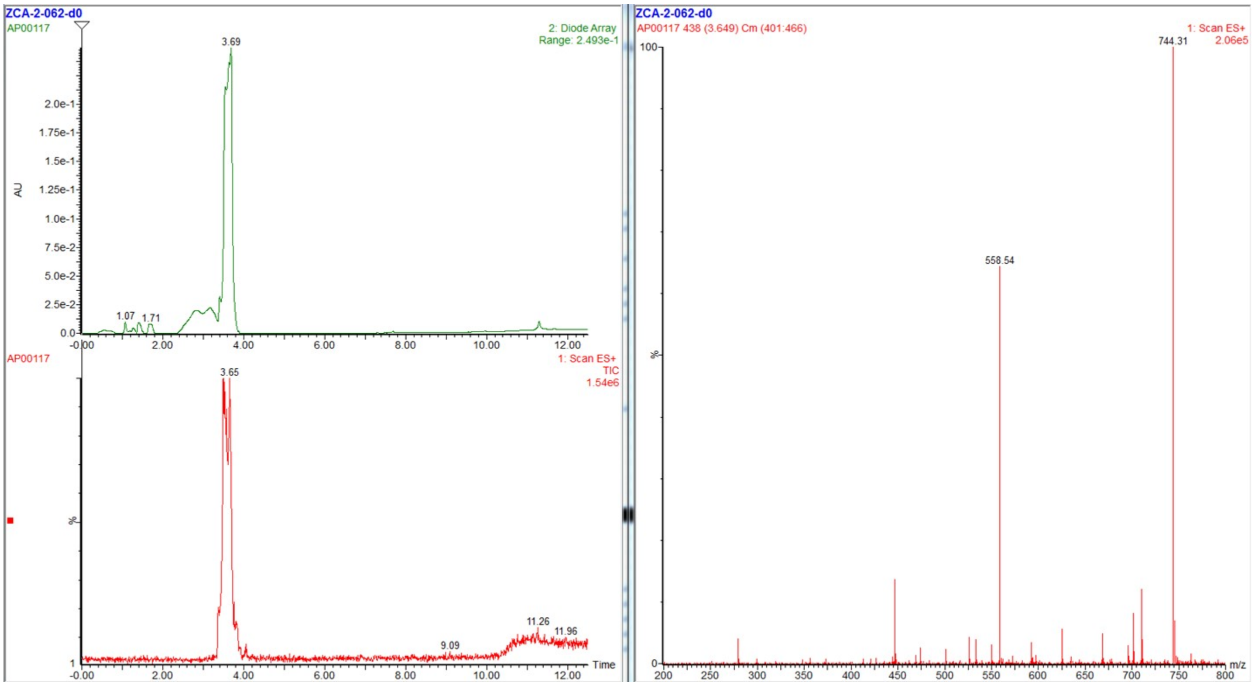

Fig. S12 LC/MS characterization of pure MAX1. Expected molecular weight 2230.0. Observed mass 2229.6. ~89% yield by UV

H—dV-dK-dV-dK-dV-dK-dV-dK-dV-P—dP-dT-dK-dV-dK-dV-dK-dV-dK-dV—NH_2

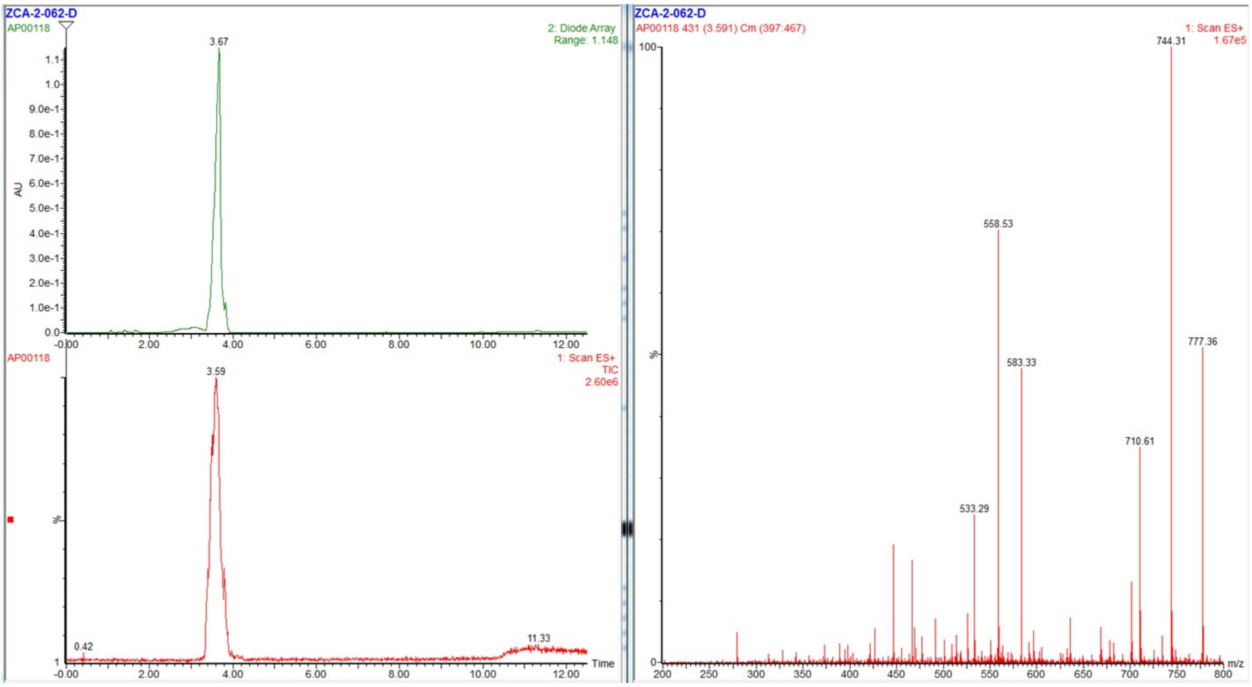

Fig. S13 LC/MS characterization of pure D-MAX1. Expected molecular weight 2230.0. Observed mass 2229.6. ~96% yield by UV

1. B. Ozbas, J. Kretsinger, R. Karthikan, J. P. Schneider and D. J. Pochan, *Macromolecules*, 2004, **37**, 7331-7337.
